# Combined quantitative tuberculosis biomarker model for time-to-positivity and colony forming unit to support tuberculosis drug development

**DOI:** 10.3389/fphar.2023.1067295

**Published:** 2023-03-14

**Authors:** Rami Ayoun Alsoud, Robin J. Svensson, Elin M. Svensson, Stephen H. Gillespie, Martin J. Boeree, Andreas H. Diacon, Rodney Dawson, Rob E. Aarnoutse, Ulrika S. H. Simonsson

**Affiliations:** ^1^ Department of Pharmaceutical Biosciences, Uppsala University, Uppsala, Sweden; ^2^ Department of Pharmacy, Uppsala University, Uppsala, Sweden; ^3^ Department of Pharmacy, Radboud Institute for Health Sciences, Radboud University Medical Center, Nijmegen, Netherlands; ^4^ Division of Infection and Global Health, School of Medicine, University of St Andrews, St Andrews, United Kingdom; ^5^ Department of Lung Diseases, Radboud University Medical Center, Nijmegen, Netherlands; ^6^ TASK Applied Science, Cape Town, South Africa; ^7^ Division of Pulmonology, Department of Medicine, University of Cape Town, Cape Town, South Africa; ^8^ University of Cape Town Lung Institute, Cape Town, South Africa

**Keywords:** rifampicin, TTP, CFU, tuberculosis, biomarker

## Abstract

Biomarkers are quantifiable characteristics of biological processes. In *Mycobacterium tuberculosis*, common biomarkers used in clinical drug development are colony forming unit (CFU) and time-to-positivity (TTP) from sputum samples. This analysis aimed to develop a combined quantitative tuberculosis biomarker model for CFU and TTP biomarkers for assessing drug efficacy in early bactericidal activity studies. Daily CFU and TTP observations in 83 previously patients with uncomplicated pulmonary tuberculosis after 7 days of different rifampicin monotherapy treatments (10–40 mg/kg) from the HIGHRIF1 study were included in this analysis. The combined quantitative tuberculosis biomarker model employed the Multistate Tuberculosis Pharmacometric model linked to a rifampicin pharmacokinetic model in order to determine drug exposure-response relationships on three bacterial sub-states using both the CFU and TTP data simultaneously. CFU was predicted from the MTP model and TTP was predicted through a time-to-event approach from the TTP model, which was linked to the MTP model through the transfer of all bacterial sub-states in the MTP model to a one bacterial TTP model. The non-linear CFU-TTP relationship over time was well predicted by the final model. The combined quantitative tuberculosis biomarker model provides an efficient approach for assessing drug efficacy informed by both CFU and TTP data in early bactericidal activity studies and to describe the relationship between CFU and TTP over time.

## 1 Introduction

Tuberculosis (TB), a bacterial infection caused by *Mycobacterium tuberculosis* (Mtb), is among the top causes of death worldwide and the second leading cause of death due to infection after COVID-19 (Global tuberculosis report 2022). New antibiotics are urgently needed due to resistance development to many existing drugs. In order to develop new antibiotics and regimens, innovative tools are needed in early development together with biomarkers which quantify the biological processes as a response to drug efficacy.

In TB drug development, early bactericidal activity (EBA) in 2-week treatment trials of TB patients are often the first assessment of drug efficacy. Colony forming unit (CFU) and time-to-positivity (TTP) are the two commonly used biomarkers in EBA studies but also in longer Phase 2b trials. Traditionally, EBA has been assessed using each of the two biomarkers where TTP often nowadays is the primary endpoint (Diacon and Donald, 2014). TTP is quantified in liquid media, often in mycobacterial growth indicator tube (MGIT). With time, and as the bacteria grow within the MGIT system, oxygen is depleted and carbon dioxide is produced, resulting in a fluorescence signal where the time to achieve the positive signal is defined as TTP. Whereas CFU only quantifies actively multiplying bacteria on solid media, it has been shown that non-multiplying bacteria can grow in liquid media (Dhillon et al., 2014), and is thought to be the more sensitive of the two (Diacon et al., 2012). The presence of non-multiplying bacteria at the end of treatment is the cause of relapse, as they act as a pool from which multiplying bacteria emerge to cause recurrent disease (Chao and Rubin, 2010). Non-multiplying bacteria most likely exists in different forms in a spectrum from truly non-multiplying to different states of multiplying forms (Tuomanen, 1986; Coates and Hu, 2008).

A study on clinical sputum samples supplemented with resuscitation-promoting factors (rpfs) showed that non-multiplying bacteria constitute the vast majority of the bacterial population pre-treatment and that CFU, as a biomarker of the multiplying bacteria, only quantifies a small proportion of the total bacterial burden in patient samples (Mukamolova et al., 2010). Bowness et al. (2015) studied the relationship between CFU and TTP using data from patients on rifampicin monotherapy through means of linear regression of data per observation day and where the gradient of the regression line and *y*-intercept of the TTP-CFU relationship increased as treatment progressed. This resulted in two samples with identical CFU readings having different TTPs if the samples were collected at different times during treatment which was suggested to be due to that TTP captures an additional sub-population that is not captured in the CFU count.

The different bacterial states can be simplified theoretically and mathematically as fast-, slow- and non-multiplying TB sub-states which have been described by the semi-mechanistic multistate tuberculosis pharmacometric (MTP) model (Clewe et al., 2016). The MTP model was developed using CFU counts from natural growth data of Mtb in an *in vitro* hypoxia system together with the decline in CFU counts in response to treatment in log and stationary phase cultures. The MTP model was since successfully used to describe other *in vitro* systems (Chen et al., 2018; Susanto et al., 2020), and was validated and used in *in vivo* settings (Chen et al., 2017; Clewe et al., 2020), and in clinical settings (Svensson and Simonsson, 2016; Faraj et al., 2020a; Faraj et al., 2020b) to predict the changes in the numbers of bacteria in the different sub-states and subsequently CFU with and without treatment. Likewise, time-to-event approaches for describing drug efficacy using TTP biomarker data in clinical trials have been developed (Chigutsa et al., 2013; Svensson and Karlsson, 2017; Svensson et al., 2018b).

In this work, we aimed to develop a combined quantitative TB biomarker model using CFU and TTP biomarker data for assessing drug efficacy in early bactericidal activity studies and to describe the relationship between CFU and TTP over time.

## 2 Materials and methods

### 2.1 Patients and data

Clinical trial data was obtained from the PanACEA HIGHRIF1 trial, an open-label phase 2a trial registered at www.clinicaltrials.gov (NCT01392911) (Boeree et al., 2015). The trial was approved by local Ethical Review Boards and by the Medical Control Council of South Africa and was conducted according to Good Clinical Practice. All patients provided written informed consent before enrollment into the study. Newly diagnosed, pulmonary TB patients, susceptible to isoniazid and rifampicin were randomized to six cohorts assigned to 10 (*n* = 8, reference arm), 20, 25, 30, 35, or 40 (*n* = 15/arm) mg/kg daily oral rifampicin monotherapy for the first 7 days. The following seven days, therapy was supplemented with isoniazid, pyrazinamide and ethambutol at standard doses. Sputum samples were collected overnight over a 16-h interval on two consecutive days at baseline and daily for one week. On each day, two replicates from each sample were cultured on agar plates to assess CFU over time, while two other replicates were cultured in liquid media to determine the change in TTP over time. The limit of quantification (LOQ) for log CFU was 1 mL^−1^, while it was 42 days for TTP samples. CFU samples below LOQ and TTP samples above LOQ were considered negative. One positive replicate was considered enough to include the sample in this analysis regardless of the other replicate as long as the next sample was positive.

In this analysis, data of daily sputum samples from a total of 83 patients on rifampicin monotherapy over the first week were included in the analysis. Contaminated CFU (*n* = 22) and TTP (*n* = 14) samples were excluded from the analysis. A total of 40 CFU samples were negative in that period and were removed from the analysis. This is because in all but two individuals, the negative samples were followed by positive CFU samples, and for those two individuals, the negative samples took place on day seven. A total of three negative TTP samples were excluded from the analysis. There were only seven instances in the CFU data and two in the TTP data when only one replicate was positive and the other was negative. Accordingly, 681 and 727 samples of CFU and TTP were included in the analysis, respectively. A description of the data and patient characteristics are summarized in Supplementary Tables S1, S2, respectively, in the Supplementary Materials.

### 2.2 Modelling strategy

In order to evaluate rifampicin exposure-response relationship using CFU and TTP, a previously developed rifampicin pharmacokinetic (PK) model using the PK data from this study was used (Svensson et al., 2018a). An individual pharmacokinetic parameter (IPP) approach (Zhang et al., 2003) was used where the individual PK model estimates were used as input for the exposure-response analysis for the CFU and TTP data. Initially, the MTP model (Clewe et al., 2016), developed to describe clinical CFU data after rifampicin treatment (Svensson and Simonsson, 2016), was used as a stand-alone model to describe only the CFU data. Thereafter, the combined quantitative TB biomarker model was developed by linking the MTP model to a TTP model. The TTP model was based on a semi-mechanistic time-to-event model previously developed using TTP data from the same clinical trial (Svensson et al., 2018b).

The MTP model had a central role within the combined quantitative TB biomarker model and acted as the link between the CFU and TTP data. While the sum of the fast- and slow-multiplying bacteria in the MTP model was used to predict CFU, the sum of all bacterial sub-states in the MTP model at the end of each sampling day, were transferred to the bacterial population in the TTP model, which thereby initiated the 0–42-day TTP. The TTP model related the mycobacterial growth in the liquid medium to the probability of achieving a positive signal in the MGIT system using a hazard model, treating the TTP observations as time-to-event data (Svensson et al., 2018b). Both CFU and TTP data were simultaneously analyzed to investigate the drug exposure-response relationship on each of the mycobacterial sub-states using the MTP model.

Once developed, the combined quantitative TB biomarker model was applied to predict one biomarker using information from the other. In order to predict the median tendency of TTP from CFU, only the CFU data were used to derive the empirical Bayes estimates (EBEs) of the final model. The opposite was applied when predicting the median tendency of CFU using only TTP data to derive the EBEs of the final model.

### 2.3 MTP model

The MTP model is a semi-mechanistic pharmacometric model, which describes the different mycobacterial sub-states and allows for the exploration of the exposure-response on those sub-states (Clewe et al., 2016). It consists of three bacterial sub-states: Fast- (F), slow- (S), and non-multiplying (N). The MTP model is represented by Eqs 1–3, in which time was defined as the time since infection.

$$\frac{dF}{dt}=Growth∙F∙F_{G}+k_{SF}∙S-k_{FS}∙F-k_{FN}∙F-F_{D}∙F$$ (1)

$$\frac{dS}{dt}=k_{FS}∙F+k_{NS}∙N-k_{SN}∙S-k_{SF}∙S-S_{D}∙S$$ (2)

$$\frac{dN}{dt}=k_{SN}∙S+k_{FN}∙F-k_{NS}∙N-N_{D}∙N$$ (3)

where $F_{G}$ , $F_{D}$ , $S_{D}$ , and $N_{D}$ are the estimated drug effects as inhibition of the fast-multiplying growth and killing of the fast-, slow-, and non-multiplying sub-states, respectively. The transfer rates between the three different sub-states are described by $k_{SF}$ , $k_{FS}$ , $k_{FN}$ , $k_{NS}$ , and $k_{SN}$ .

Only the fast-multiplying sub-state was assumed to grow, with the growth limited by the system carrying capacity parameter ( $B_{\max}$ ). The growth of slow-multiplying sub-state was not included, and the increase of slow-multiplying sub-state took place through the transfer of bacteria from the fast-and non-multiplying state (Clewe et al., 2016). The transfer rates between the three different sub-states were fixed to the *in vitro* estimates (Clewe et al., 2016), as it was not possible to estimate such system-specific parameters separately from drug exposure-response parameters using the current data of rifampicin treatment. In addition, the transfer rate from the non-multiplying bacterial sub-state to the fast-multiplying bacterial sub-state was considered negligible based on the previous publication (Clewe et al., 2016). Furthermore, the transfer rate from the fast-to the slow-multiplying sub-states ( $k_{FS}$ ) increased linearly with time. A Gompertz growth function was chosen to describe the growth of the fast-multiplying sub-state:

$$Growth=k_{G}∙\log\left(\frac{B_{\max}}{F+S+N}\right)$$ (4)

where $k_{G}$ is the growth rate of the fast-multiplying bacterial sub-state in the MTP model and $B_{\max}$ is the system carrying capacity, which defines the bacterial at start of treatment.

In this study, patients were assumed to be in the stationary phase of the infection, which is characterized by stable bacterial counts over time in untreated patients (Jindani et al., 1980). Patients were, thus, assumed to start treatment 150 days after the TB infection, a time-point at which the MTP model predicts a negligible change in the bacterial population, and little to no growth is expected. As the data was not sufficient to the estimate the acute bacterial growth phase, the $k_{G}$ parameter was fixed to the *in vitro* estimate (Clewe et al., 2016). In addition, the initial conditions occurring 150 days before baseline, i.e., at time of infection, were fixed to zero for the non-multiplying stub-state and to 4.10 and 9,770 mL^−1^ for the fast- and slow-multiplying sub-states, respectively, based on the original work and its clinical application (Clewe et al., 2016; Svensson and Simonsson, 2016). On the other hand, the $B_{\max}$ parameter was re-estimated, along with its inter-individual variability (IIV), to account for the different individual bacterial baselines without affecting the relative amounts of the bacterial populations.

From the MTP model, the bacterial sub-states were transferred to the TTP model, while CFU was predicted as the sum of only the fast- and slow-multiplying sub-states using Eq 5.

$$\log_{10}CFU=\log_{10}\left(F+S\right)$$ (5)

where log_10_ CFU is the logarithm of CFU at a specific time-point, and F and S are the fast- and slow-multiplying sub-states, respectively, at that same time-point.

### 2.4 TTP model

The TTP model was based on an earlier published TTP model (Svensson et al., 2018b). The model describes the growth of the bacterial liquid culture from patients’ sputum samples. The starting bacterial load in the tube was evaluated in different ways such as a one subpopulation model and a three-subpopulation model (i.e., MTP model structure). In addition, the use of a correction factor ( CF ) that scaled down the ratio of the non-multiplying bacteria to the total bacteria that is transferred from the MTP model to the tube bacterial compartment was also evaluated ( F+S+N∙CF) . The CF parameter was both estimated and fixed to 17% (Faraj et al., 2020a). This is because it has been previously shown that the ratio of the non-multiplying sub-state to the total bacteria had to be scaled down to correctly predict the clinical bacterial number using an MTP model developed using *in vitro* data (Faraj et al., 2020a).

In the TTP model, another time scale was used, which was time since inoculation in liquid media. The total bacteria in the bacterial compartment were assumed to give rise to the signal in the MGIT system, as it was expected that, once introduced into the fresh media in the tube, the non-multiplying bacteria will re-initiate protein synthesis (Hu et al., 1998) and contribute to the total bacterial growth. A liquid culture-specific system carrying capacity parameter ( $B_{\max,lc}$ ) and a liquid culture-specific growth rate constant ( $k_{G,lc}$ ), both controlling the growth of the bacterial liquid culture, were estimated. This is because the bacterial population is expected to grow differently in the MGIT system, as a growth enhancing substance is added to the MGIT liquid medium to reduce the detection time (Siddiqi and Rüsch-Gerdes, 2006). Different models of growth in the liquid medium were evaluated, including exponential, logistic and Gompertz growth functions.

Sputum samples undergo dilution and centrifugation steps during processing that remove any antibiotics that might have been present in the samples before being placed in the liquid medium. Consequently, the TTP model did not include killing by drug. Taking into consideration that post antibiotic effect (PAE) might take place within the liquid culture, different lag-time models in addition to time-varying growth rate in the liquid culture were evaluated to describe a potential delay or change of bacterial growth after different drug exposures. Furthermore, IIV, reflecting variability in the bacterial metabolic activity, was explored for the different liquid culture parameters.

As the TTP model describes time-to-event data, a hazard model, which described the probability of achieving a positive signal in the MGIT system, was employed, with right censoring occurring on day 42. At any given time-point in the liquid culture ( $t_{lc}$ ), the hazard, $h\left(t_{lc}\right)$ , was calculated and was equal to the total bacterial load multiplied by a hazard scaler ( HS ) as:

$${h(t}_{lc})={B_{lc}(t}_{lc})∙HS$$ (6)

where $\left.{h(t}_{lc}\right)$ is the hazard at one time-point, $\left.{B_{lc}(t}_{lc}\right)$ is the total bacterial liquid culture at that time-point, and HS is a scaling parameter for the hazard.

The hazard at each time point was integrated to calculate a cumulative hazard, $\left.{H(t}_{lc}\right)$ :

$${H(t}_{lc})=\overset{t_{lc}}{\underset{0}{\int }}\left.{h(t}_{lc}\right)\;dt$$ (7)

where $\left.{H(t}_{lc}\right)$ is the cumulative hazard at time in the liquid culture and $\left.{h(t}_{lc}\right)$ is the hazard at that time-point.

Finally, the survival, which describes the probability of not yet observing a positive signal was:

$${S(t}_{lc})=e^{\left.-{H(t}_{lc}\right)}$$ (8)

where $\left.{S(t}_{lc}\right)$ is the survival at time in the liquid culture and $\left.{H(t}_{lc}\right)$ is the cumulative hazard at that time-point.

### 2.5 Exposure-response relationships

A previously developed rifampicin PK model by Svensson et al. (2018a) was linked to the MTP model. Different exposure-response relationships were evaluated on four different effect sites of the MTP model in four steps using the combined quantitative TB biomarker model and both CFU and TTP data. The different exposure response relationships that were evaluated were on/off, linear, $E_{max}$ , and sigmoidal $E_{\max}$ relationships. The four different effect sites that were evaluated were inhibition of the growth of the fast-multiplying sub-state ( $F_{G}$ ) and killing of each of the fast- ( $F_{D}$ ), slow ( $S_{D}$ ), and non-multiplying ( $N_{D}$ ) sub-states. The first step was to evaluate all exposure-response relationships on all effect sites in a univariate approach. The models were considered statistically significant at a significance level of 5%, i.e., drop in objective function value (OFV) of at least 3.84, for nested models with one additional parameter. The second step involved evaluating the statistically significant exposure-response models kept from the univariate evaluation of each effect site in combinations of two, three, and then all four effects sites, with at least a linear model on each site. The third step involved re-evaluating the exposure-response relationship at each effect site, and only statistically significant models were kept. The model with the largest OFV drop was selected and evaluated in combination with the model with the second highest significant drop until no significant drop in OFV was observed. The fourth step involved removing one exposure-response relationship from each effect site. In this backward elimination step, a significance level of 1%, i.e. 6.63 OFV drop, was used. After applying the four steps, the model with the final exposure-response relationship was obtained. IIV on the different exposure-response parameters were also evaluated.

### 2.6 Software and model selection

The CFU and TTP data analyses were performed using NONMEM 7.4.3 (Icon Development Solutions, Elliott City, MD, United States) (Beal et al., 2018) with the Laplacian estimation method. Data management and visualization were done in R statistical software version 4.1.2 (R Foundation for Statistical Computing, Vienna, Austria) (R Development Core Team, 2017). PsN 5.0.0 (Lindbom et al., 2005) was used to run the models and produce visual predictive checks (VPCs) used for model diagnostics (Lindbom et al., 2005). Additional graphical assessments of results were performed in xpose 4.7.2 (Keizer et al., 2013).

Model evaluation was based on parameter uncertainty and scientific plausibility while they were visually assessed using goodness-of-fit plots and VPCs. Nested models were evaluated based on the OFV, using the likelihood ratio test at a 5% significance level. For CFU, conventional VPCs, comparing observed and simulated data stratified by dose group, were produced. For TTP, posterior predictive checks (PPC) of the TTP in days versus time since treatment, stratified by each dose group, were used. Sampling importance resampling (SIR) was used to obtain accurate parameter uncertainties (Dosne et al., 2016).

## 3 Results

**FIGURE 1:**
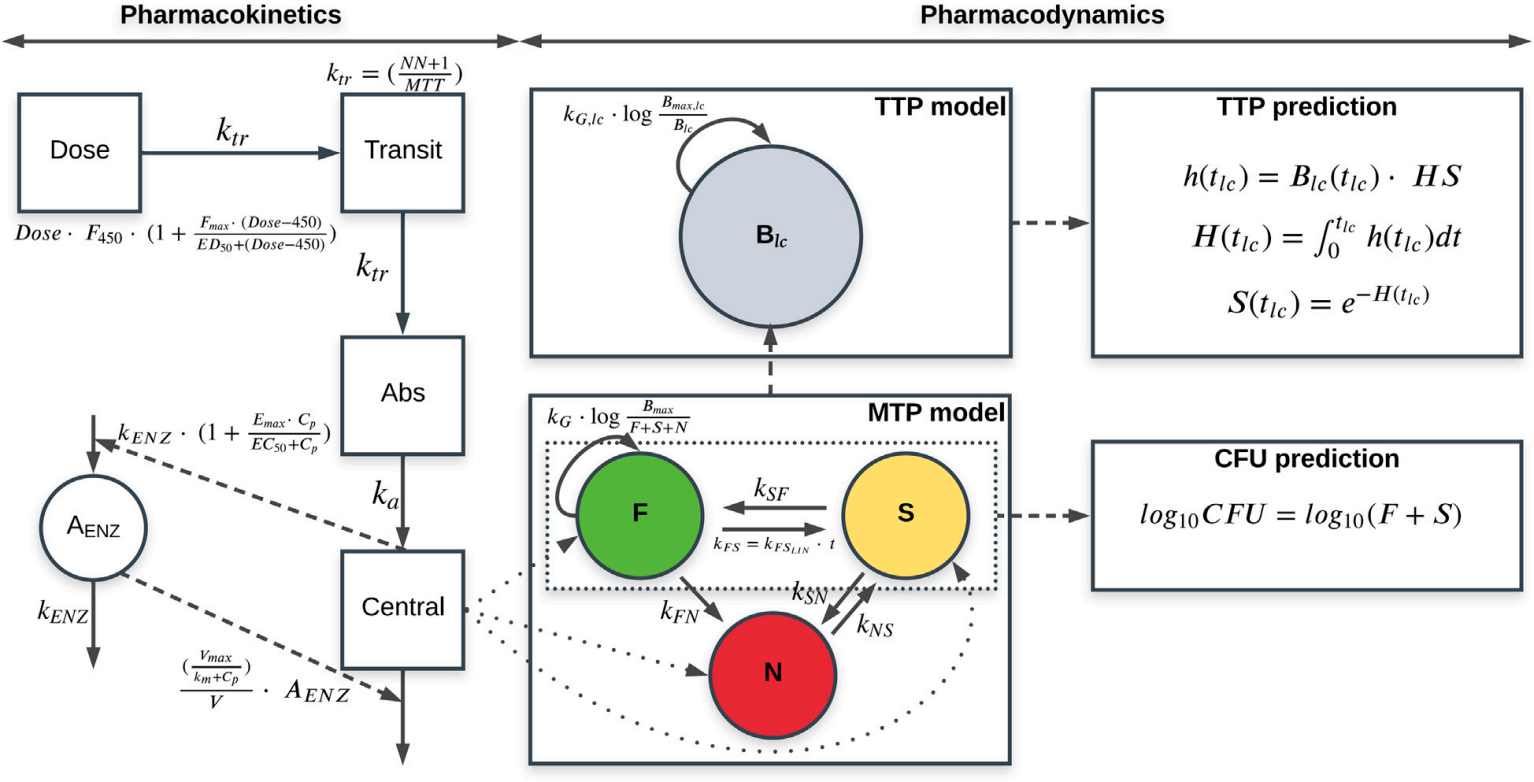
Schematic representation of the final quantitative tuberculosis biomarker model, including the pharmacokinetic (PK) model (left), MTP model (lower middle) and time-to-positivity (TTP) model (upper middle) using early bactericidal activity data of rifampicin in monotherapy in tuberculosis patients. CFU and TTP observations are simultaneously informing the MTP and TTP models through the simultaneous fit of the model to the data. The MTP model is represented by different bacterial sub-states; fast- (F), slow (S) and non-multiplying sub-state (N). The PK model is linked to the MTP model through the different killing parameters. CFU is predicted from the MTP model as the sum of the F and S sub-states. The TTP model consists of one bacterial sub-population model ( $B_{lc}$ ) which is the sum of the F, S, and N sub-states at the starting time in the liquid medium ( $t_{lc}$ = 0). The bacterial liquid culture at any given time point in the liquid medium $\left[{B_{lc}(t}_{lc}\right)$ ] was equal to the hazard $\left.{h(t}_{lc}\right)$ multiplied by a hazard scaler ( HS ). The probability of a TTP sample without a positive signal at time $t_{lc}$ was given by the survival [ $\left.{S(t}_{lc}\right)$ ] which was derived from the cumulative hazard [ $\left.\left.{H(t}_{lc}\right)\right]$ . $F_{450}$ , relative bioavailability to 450 mg; $F_{\max}$ , maximal increase in relative bioavailability; ${ED}_{50}$ , dose corresponding to half the $F_{\max}$ ; NN , number of transit compartments; $k_{tr}$ , transfer rate between transit compartments; MTT , mean transit time; $k_{a}$ , absorption rate constant; $k_{ENZ}$ , first-order enzyme degradation rate and zero-order enzyme formation rate; $E_{\max}$ , maximal increase in enzyme formation rate; ${EC}_{50}$ , concentration corresponding to 50% of $E_{\max}$ ; $C_{p}$ , plasma concentration; $V_{\max}$ , the maximal elimination rate; $k_{m}$ , $C_{p}$ at half of $E_{\max}$ ; V , volume of distribution; $k_{G}$ , growth rate of the fast-multiplying state; $B_{\max}$ , system carrying capacity per mL^-1^; $k_{FSLin}$ , time-dependent linear rate parameter describing transfer from fast-to slow-multiplying states; $k_{SF}$ , first-order transfer rate from slow-to fast-multiplying states; $k_{FN}$ , first-order transfer rate between fast- and non-multiplying states; $k_{SN}$ , first-order transfer rate between slow- and non-multiplying states; $k_{NS}$ , first-order transfer rate between non- and slow-multiplying states; $B_{lc}$ , total bacterial liquid culture; $B_{\max,lc}$ , system carrying capacity per mL^-1^ in liquid media; $k_{G,lc}$ , growth rate of the bacterial liquid culture; HS , hazard scaler; t , time since infection; $t_{lc}$ , time in liquid culture. Solid arrows represent mass transfer, while dashed arrows represent linking between models.

The general structure of the final quantitative TB biomarker model consisted of one PK model and two biomarker models: the MTP and TTP models (Figure 1).

All MTP model parameters were fixed to the *in vitro* estimates (Clewe et al., 2016), except for $B_{\max}$ , which was estimated. Estimating IIV on $B_{\max}$ was necessary to allow for the prediction of the individual bacterial load at baseline. The MTP model previously employed by Svensson and Simonsson (2016) to describe clinical CFU data, was first applied to CFU data as a stand-alone model. The model described the CFU data well, which provided a validation for using the MTP model in this work.

The TTP model comprised a one bacterial population that was initialized as the sum of the fast-, slow-, and non-multiplying sub-states in the MTP model at the sampling occasion. An MTP model structure within the TTP model, representing all three sub-states in the liquid medium with all transfer rates between the sub-states fixed to the *in vitro* estimates, was also evaluated, in which the positive signal in the MGIT system was driven by the sum of the fast-, slow-, and non-multiplying sub-states. However, the TTP data did not support the MTP model structure within the TTP model. Therefore, a one bacterial liquid culture ( $B_{lc}$ ) was explored and shown to describe the data:

$$\frac{{dB}_{lc}}{dt_{lc}}={Growth}_{lc}∙B_{lc}$$ (9)

where ${Growth}_{lc}$ and $B_{lc}$ are the liquid culture-specific growth and total bacterial liquid culture, respectively.

Adding a fixed CF parameter ( CF = 17%) to correct for the amount of the non-multiplying sub-state transferred from the MTP model to the TTP compartment resulted in a higher OFV, while estimating it did not improve the model fit to the data, and thus, the CF parameter was not used. Instead, the total sum of all bacterial sub-states ( F+S+N) was transferred from the MTP model to the liquid medium in the TTP model.

The liquid culture-specific growth was described by a Gompertz growth model as it improved model fit compared to logistic and exponential growth models. Estimating a liquid culture-specific bacterial growth rate ( $k_{G,lc}$ ) was necessary and resulted in a 314-point drop in OFV compared to using the fixed *in vitro* $k_{G}$ parameter. This is in line with the expectation of the different bacterial growth in the MGIT system due to the added growth enhancing substance in the liquid medium (Siddiqi and Rüsch-Gerdes, 2006). Furthermore, a liquid culture-specific $B_{\max,lc}$ , controlling the growth in the liquid medium, was estimated and provided a significantly better fit to the data with a 629 drop in OFV compared to using the *in vitro* $B_{\max}$ parameter. A time-varying mycobacterial growth and a delayed liquid culture growth did not statistically significantly decrease the OFV. Eq.10 shows the Gompertz growth model in the TTP model.

$$Growth_{lc}=k_{G,lc}\cdot \log\left(\frac{B_{\max,lc}}{B_{lc}}\right)$$ (10)

where $k_{G,lc}$ is the liquid culture growth rate, $B_{\max,lc}$ is system carrying capacity, and $B_{lc}$ is the total bacterial liquid culture.

Using the MTP model approach, a linear effect described the killing of the fast-multiplying sub-state, while an $E_{\max}$ effect described the killing of each of the slow- and non-multiplying sub-states. No further significant OFV drop was seen when evaluating more complex models, e.g., $E_{\max}$ for the killing of the fast-multiplying sub-state or sigmoidal $E_{\max}$ model the killing of either the slow- and non-multiplying sub-states. Additionally, no statistically significant exposure-response relation was found on the inhibition of growth of the fast-multiplying sub-state. IIV was evaluated on all exposure-response parameters but was not supported by the data. The exposure-response relationships identified using both CFU and TTP data were different compared to fitting the CFU stand-alone model to the CFU data. While both models identified an $E_{\max}$ relationship on the killing of the slow- and non-multiplying sub-states, using only CFU data identified a simpler on/off model for the inhibition of growth of the fast-multiplying sub-state.

**FIGURE 2:**
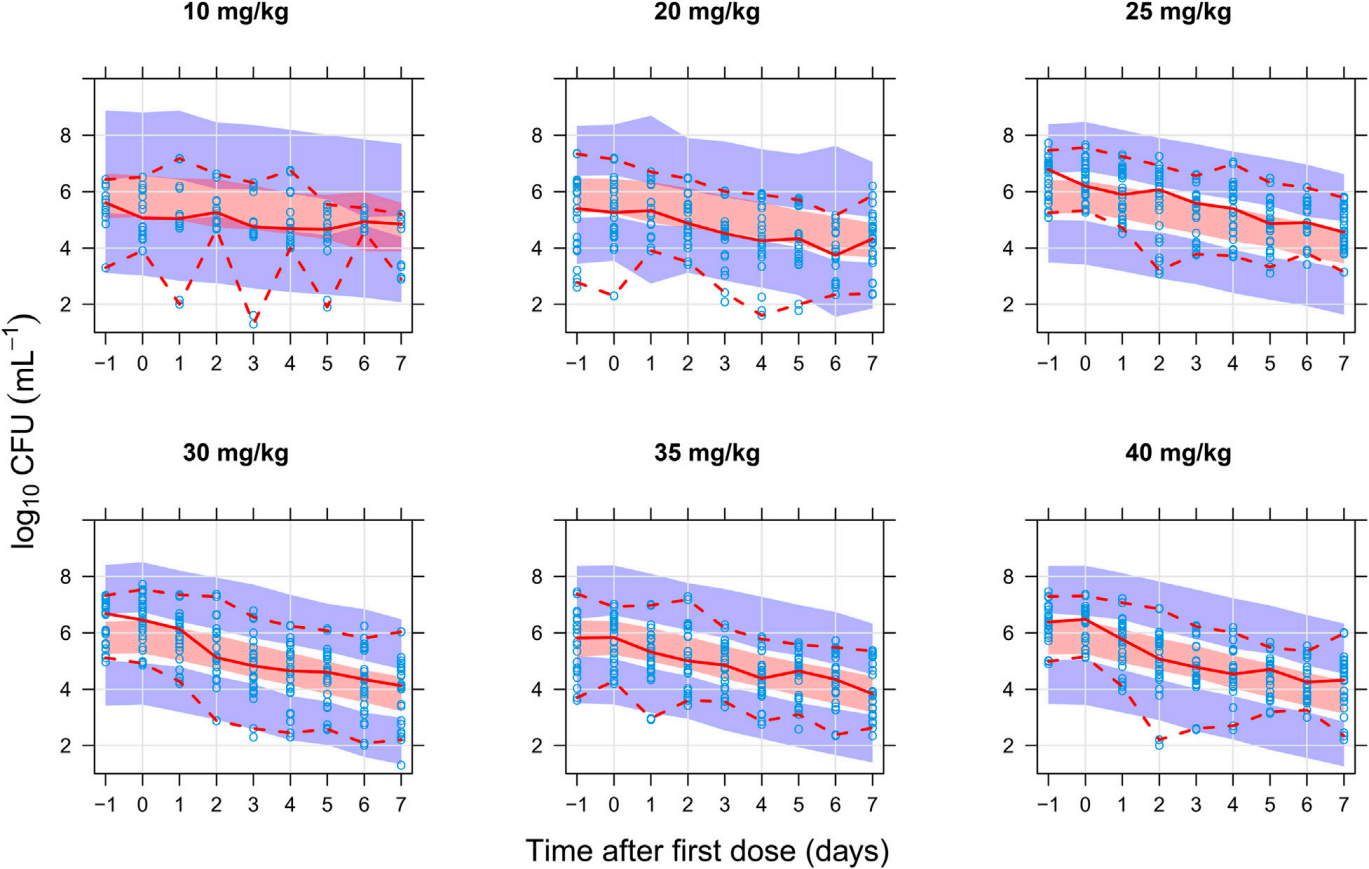
Visual predictive check of the final quantitative biomarker model describing colony forming unit (CFU) versus time since first dose and stratified on rifampicin dose group. The solid and dashed lines are the median, 2.5th, and 97.5th percentiles of the observed data, respectively. The shaded areas are the 95% confidence intervals of the 97.5th (blue), median (red), and 2.5th (blue) percentiles of the simulated data based on 1,000 simulations. Open circles are the observations.

**FIGURE 3:**
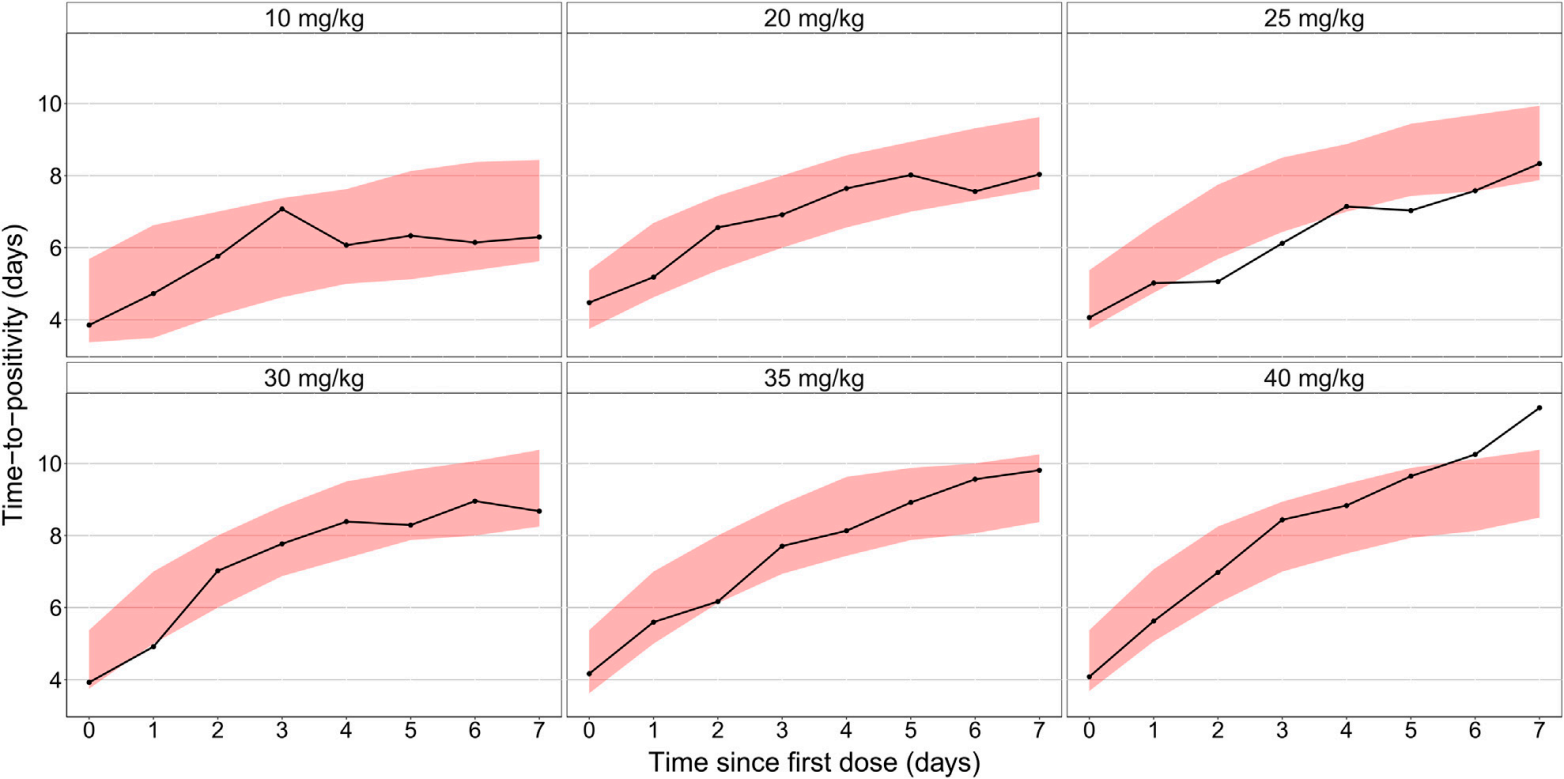
Posterior predictive check of the final quantitative biomarker model describing median time-to-positivity (TTP) versus time since first dose and stratified per rifampicin dose group. Solid lines represent the median time to positivity based on the observed data. Shaded areas are the 90% prediction interval based on 1,000 simulations using the model.

The final combined quantitative TB biomarker model provided a good fit to both the CFU and TTP data as seen in the VPC of CFU versus time stratified by dose group (Figure 2) and the PPC of TTP versus time (Figure 3), respectively. A Kaplan-Meier VPC of TTP versus time in the liquid medium for different rifampicin doses and treatment days is included in Supplementary Figure S1. The final parameter estimates from the combined biomarker model is shown in Table 1.

**TABLE 1:** Final quantitative tuberculosis biomarker model parameter estimates.

Parameter	Description	Estimate^a^	RSE%^b^
*MTP model*			
$k_{G}$ (days^-1^)	Fast-multiplying bacterial growth rate	0.206 FIX	-
$k_{FSLin}$ (days^-1^)	Time-dependent transfer rate from fast- to slow-multiplying state	0.00166 FIX	-
$k_{FN}$ (days^-1^)	Transfer rate from fast- to non-multiplying state	8.97∙10^−7^ FIX	-
$k_{SF}$ (days^-1^)	Transfer rate from slow- to fast-multiplying state	0.0145 FIX	-
$k_{SN}$ (days^-1^)	Transfer rate from slow- to non-multiplying state	0.186 FIX	-
$k_{NS}$ (days^-1^)	Transfer rate from non- to slow-multiplying state	0.00123 FIX	-
$B_{\max}$ (mL^-1^)	System carrying capacity	4.997∙10^4^	5.27
$F_{0}$ (mL^-1^)	Initial bacterial number of fast-multiplying state	4.1 FIX	-
$S_{0}$ (mL^-1^)	Initial bacterial number of slow-multiplying state	9770 FIX	-
IIV $B_{\max}$ (%)	Inter-individual variability in $B_{\max}$	83.4	12.1
ε (CV%)	Additive residual error on log scale	59.4	4.02
$\varepsilon_{repl}$ (CV%)	Additive replicate error on log scale	19.1	24.1
*TTP model*			
$B_{\max,lc}$ (mL^-1^)	System carrying capacity per mL in liquid culture	1.62∙10^6^	7.61
$k_{G,lc}$ (day^-1^)	Mycobacterial growth rate in liquid culture	0.22	4.34
HS	Hazard scaler	1.49∙10^−6^	6.99
*Exposure-response*			
$FD_{k}$ (L·mg^-1^·days^-1^)	Second-order fast-multiplying state death rate	16.5	333
$S{D_{E}}_{\max}$ (days^-1^)	Maximum achievable drug-induced slow-multiplying state kill rate	0.30	18.3
$S{D_{EC}}_{50}$ (mg·L^-1^)	Concentration at 50% of $S{D_{E}}_{\max}$	24.9	36.5
$N{D_{E}}_{\max}$ (days^-1^)	Maximum achievable drug-induced non-multiplying state kill rate	1.93	10.8
$N{D_{EC}}_{50}$ (mg·L^-1^)	Concentration at 50% of $N{D_{E}}_{\max}$	4.83	30.5

^a^ All fixed parameters in this model were obtained from Clewe et al. (2016), and applied in Svensson and Simonsson (2016).

^b^ RSE, relative standard error reported on the approximate standard deviation scale obtained using sampling importance resampling (SIR) (Dosne et al., 2016).

**FIGURE 4:**
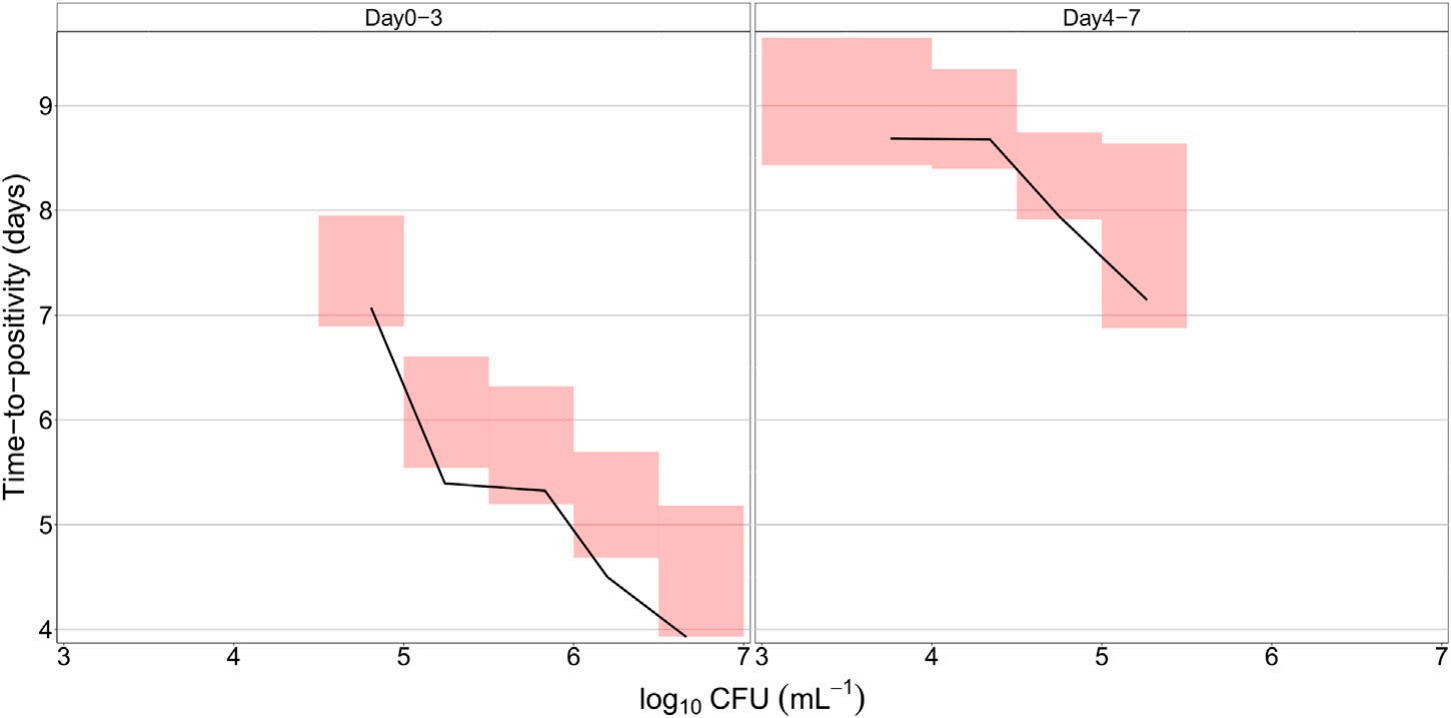
Predicted relationship between colony forming unit (CFU) versus time-to-positivity (TTP) using the final quantitative biomarker model. The solid lines represent the median of the observed data, while the shaded areas outline the 95% confidence interval based on 1,000 simulations.

**FIGURE 5:**
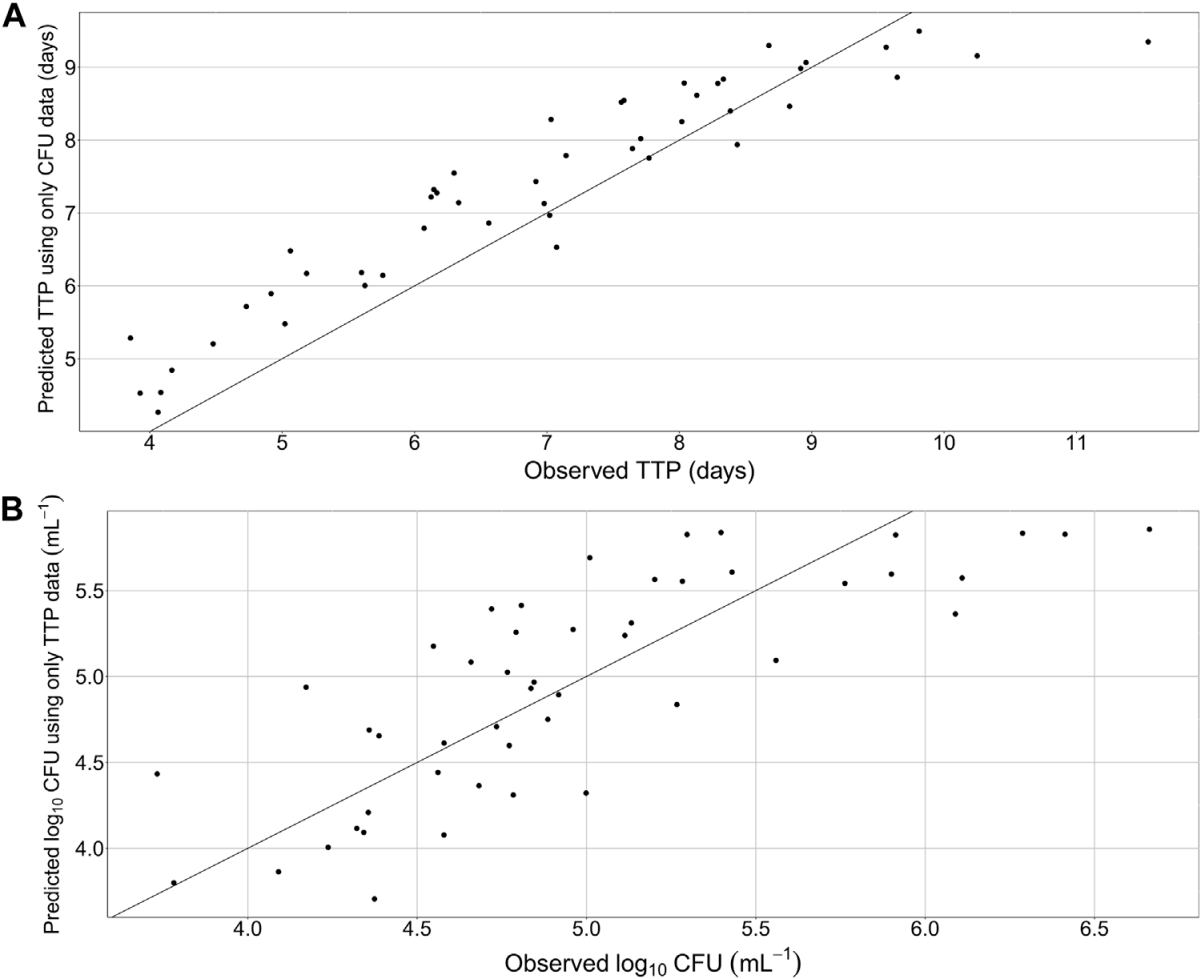
Predicted median tendency of the observed time-to-positivity (TTP) versus observed TTP, using only colony forming unit (CFU) data and the final quantitative biomarker model **(A)** and predicted median tendency of the observed CFU versus observed CFU, using only TTP data and the final quantitative biomarker model **(B)**. Solid line is the line of identity.

The final quantitative TB biomarker model was also able to describe the median CFU-TTP relationship well (Figure 4). Deriving the EBEs of the final model using data on either biomarker allowed for the prediction of the median tendency of the other biomarker. The final model was able to predict the median tendency of TTP using only CFU data with an R^2^ of 0.79 (Figure 5A). However, it was difficult for the model to predict the median tendency of CFU using only TTP data with high precision (R^2^ = 0.51; Figure 5B).

## 4 Discussion

This paper presents the development of a combined quantitative TB biomarker model (Figure 1), consisting of a PK-CFU-TTP model, to identify the relationship between CFU and TTP based on data from patients with pulmonary TB on 10–40 mg/kg rifampicin monotherapy for seven days in an early bactericidal activity (EBA) trial. The MTP model acted as the link between the CFU and TTP data within the combined model where CFU was the sum of the fast- and slow-multiplying sub-states which varied over time due to drug effect, while the total bacterial load predicted from the MTP model at the end of each day was the starting point for the level of bacteria in the TTP model.

This work builds on the MTP concept of three different sub-states; fast-, slow- and non-multiplying bacteria (Clewe et al., 2016). The MTP model has been shown to describe the growth, in the absence of treatment, and decline, in response to treatment, of bacteria in the different sub-states and, subsequently, CFU, using *in vitro* and clinical data (Clewe et al., 2016; Svensson and Simonsson, 2016). Consequently, the MTP model has been successfully applied to describe *in vitro* (Clewe et al., 2016; Susanto et al., 2020), mouse (Chen et al., 2017), and clinical data (Svensson and Simonsson, 2016; Faraj et al., 2020a; Faraj et al., 2020b). In addition, the MTP model has been successfully used to predict observations from early clinical studies using clinical dose-response forecasting from pre-clinical *in vitro* studies of rifampicin (Wicha et al., 2018; Susanto et al., 2020). Furthermore, it has been shown that applying semi-mechanistic modelling approaches, such as the MTP model, can increase the power of phase IIa studies and reduce the number of patients required to characterize drug exposure-response as compared to traditional statistical methods, e.g., *t*-test or ANOVA, and empirical approaches, e.g., mono- or bi-exponential models (Svensson et al., 2017). Applying the MTP model approach for representing bacterial burden presents a number of advantages, as representing drug efficacy on different mycobacterial sub-states is more mechanistically plausible, and it allows the evaluation of the drug exposure-response relationships on each of those populations separately, offering a mechanistic interpretation of rifampicin’s effect on different bacterial sub-states.

The EBA of rifampicin was evaluated using all available CFU and TTP data simultaneously, which further supported a strong mechanistic approach when developing the work and assessing the drug efficacy. Studies have shown that the combined use of two biomarkers better ranked the efficacy between treatments compared to using only one biomarker, as in the case of the combined use of CFU and RS ratio (Dide-agossou et al., 2022). Liquid media are known to be more sensitive in detecting the Mtb populations than solid media but are more prone to contamination (Cruciani et al., 2004; Diacon et al., 2012). International guidelines, therefore, recommend the use of at least one solid and one liquid medium to quantify the bacterial load accurately (Behr et al., 2022). As such, the simultaneous analysis of both CFU and TTP data in the combined biomarker model provided more information about the drug efficacy than only CFU, although this work only evaluated one drug. The rifampicin PK model used (Svensson et al., 2018a) accounted for the non-linearity in PK by a concentration-dependent apparent clearance and a dose-dependent relative bioavailability. The model also accounted for the decrease in rifampicin exposure with time by employing an enzyme turnover model to incorporate rifampicin’s auto-induction. The estimated exposure-response relationships included a linear killing of the fast-multiplying sub-state and a non-linear $E_{\max}$ killing for both the slow- and non-multiplying sub-states, with a higher predicted drug potency towards the non-multiplying sub-state (Table 1). In the final combined biomarker model, the precision in the rate of the killing of the fast-multiplying sub-state ( $FD_{k})$ parameter was low (Table 1). However, omitting the parameter from the model led to an increase in OFV of 174 points, and a simpler on-off model did not provide a good fit of the data. Further, omitting the parameter led to a too low predicted decline in biomarker response at day seven compared to keeping the parameter in the model.

The MTP model applied in this work is in agreement with the conclusions from Bowness et al. (2015) who also used data from the same trial, in that TTP captures an additional bacterial population that is not detected by CFU. In this work, by allowing the transfer of the sum of the bacterial sub-states in the MTP model to the TTP model, we assumed that TTP can capture the non-multiplying sub-state as the extra bacterial population that CFU does not capture, and by doing so the model is able to predict both CFU and TTP adequately well. The relationship between CFU and TTP is non-linear with respect to time and in this model approach, the non-linear relationship with time is predicted.

Earlier work has shown that the non-multiplying bacteria in sputum is 17% of the *in vitro* levels, suggesting a difference in phenotypic resistance, whereas no difference in multiplying bacteria was found (Faraj et al., 2020a). In this work, we explored the option to only allow for a percentage of the non-multiplying bacteria to be transferred to the TTP bacterial compartment from the MTP model but the data in this work did not support it, and the total bacteria from the MTP model seemed to well represent the bacterial population driving the TTP signal. A semi-mechanistic time-to-event approach was employed to describe the TTP observations in this work. While an implementation of the full MTP model with three bacterial compartments in the liquid medium was not supported by the data, a one-bacterial compartment, representing the total bacterial population in the liquid medium, was sufficient and provided a satisfactory description of the TTP data. Future work should aim at describing the dynamics of the bacterial populations in liquid media.

A stand-alone TTP model has been previously used to assess the exposure-response relationship of rifampicin using TTP data from the same trial (Svensson et al., 2018b). The stand-alone TTP model was not linked to a rifampicin PK model but instead used rifampicin exposure as a covariate on the kill rate parameters. In addition, other models have previously analyzed CFU and TTP simultaneously using EBA data. Gausi et al. (2021) used both biomarkers to describe isoniazid efficacy on a single bacterial population for CFU and TTP in patients with drug-sensitive and drug-resistant TB. Lyons used CFU and TTP to establish pretomanid and bedaquiline exposure-response relationships (Lyons, 2019; Lyons, 2022). This work builds upon the previous work by using both CFU and TTP data simultaneously combined to a rifampicin PK model to evaluate the exposure-response relationship of rifampicin on three bacterial subpopulations using the MTP model, which presents a more mechanistic approach of assessing the exposure-response relationship. Inclusion of a non-multiplying state may provide a deeper link between EBA and predictions of long-term efficacy in TB drug development although this still remains to be further investigated.

While CFU has been the gold standard biomarker in pre-clinical studies and in TB diagnosis, TTP has been suggested as a substitute to CFU in phase IIa EBA studies (Diacon et al., 2012). As such, the combined quantitative TB biomarker model developed in this work (Figure 1) can play a vital role in predicting TTP from trials where only CFU was measured. In addition, the combined quantitative TB biomarker model can be used to predict typical TTP in the case of missing or contaminated samples. In this work, the combined quantitative TB biomarker model was shown to be able to predict the median tendency of the observed TTP using information on CFU with good precision after 7-day rifampicin therapy of drug susceptible TB (Figure 5A). The model was less precise, however, in predicting the median tendency of the observed CFU using only TTP data (Figure 5B). This is because TTP was assumed to correspond to a signal initiated by the growth of the whole bacterial population. Thus, it was difficult for the model to differentiate between the different sub-states using TTP data in order to predict CFU, which was assumed to be the sum of only the fast- and slow-multiplying sub-states.

The combined quantitative TB biomarker model was developed using data from seven-day rifampicin monotherapy of drug susceptible TB (Boeree et al., 2015). As the clinical data did not include information on the acute bacterial growth phase, the growth of the different bacterial sub-states and the transfer rates between them could not be estimated. Using *in vitro* information compensates for this limitation, and thus, the growth and transfer rate parameters were fixed from the *in vitro* setting (Clewe et al., 2016). In this work, the model assumed that the TTP signal is driven by the sum of the fast-, slow-, and non-multiplying population. While several implementations for which population drives the TTP signal have been explored, the whole bacterial population driving the signal provided the best fit. The assumption that TTP can capture all bacterial populations, however, might not be true, and re-evaluation of the population driving the TTP signal might be necessary when applying the model to other data.

It has been reported that the CFU-TTP relationship might differ in certain cases of Mtb mutations (le Roux et al., 2021). As the response to treatment is expected to differ between drug-sensitive and drug-resistant Mtb strains (Bahuguna and Rawat, 2020), the model should be validated using data from treatment of drug-resistant Mtb. Potential differences in CFU-TTP relationship could be accounted for in the model to allow for a mutation specific relationship. Further work should also explore if the identified CFU-TTP relationship is drug-specific, i.e., to apply the model to different EBA clinical trial datasets. It is important to emphasize that the exposure-response relationships always are drug-specific and are better characterized using both biomarkers rather than either. Likewise, further work should also evaluate whether the identified CFU-TTP relationship holds in trials with durations longer than seven days. Therefore, extrapolation to other drugs and outside the dose range, bacterial susceptibly and treatment duration should be done with caution.

The combined rifampicin PK-linked quantitative TB biomarker model was successfully developed using CFU and TTP data from a phase IIa EBA trial. The model used data from both biomarkers to evaluate rifampicin exposure-response relationship in an EBA trial. The final model was able to describe the relationship between CFU and TTP over time.

## Data Availability

The data analyzed in this study is subject to the following licenses/restrictions: Not publicly available. Requests to access these datasets should be directed to PanACEA@radboudumc.nl.
