# Supplementary material for: Combined quantitative tuberculosis biomarker model for time-to-positivity and colony forming unit to support tuberculosis drug development
Source: Front Pharmacol. 2023 Mar 14;14:1067295. doi: 10.3389/fphar.2023.1067295 (PMC10043246; doi:10.3389/fphar.2023.1067295)
Supplement: Supplementary file 1 [file DataSheet1.docx]

Supplementary Material

# Supplementary Tables

## Table S1: Data description by rifampicin dose

|  | **Overall** | **10 mg/kg** | **20 mg/kg** | **25 mg/kg** | **30 mg/kg** | **35 mg/kg** | **40 mg/kg** |
| --- | --- | --- | --- | --- | --- | --- | --- |
| **CFU** | | | | | | | |
| **Total samples (n)** | 743 | 72 | 134 | 135 | 134 | 135 | 133 |
| **Positive samples (n [%])** | 681 (91.7) | 62 (86.1) | 106 (79.1) | 131 (97.0) | 129 (96.3) | 121 (89.6) | 132 (99.2) |
| **Negative samples (n [%])^a^** | 40 (5.4) | 3 (4.2) | 24 (17.9) | 2 (1.5) | 5 (3.7) | 5 (3.7) | 1 (0.8) |
| **Contaminated samples (n [%])** | 22 (3.0) | 7 (9.7) | 4 (3.0) | 2 (1.5) | 0 (0) | 9 (6.7) | 0 (0) |
| **Median baseline (mL^-1^)** | 6.1 | 5.43 | 5.26 | 6.46 | 6.64 | 5.83 | 6.41 |
| **TTP** | | | | | | | |
| **Total samples (n)** | 744 | 72 | 135 | 135 | 134 | 135 | 133 |
| **Positive samples (n [%])** | 727 (97.7) | 71 (98.6) | 132 (97.8) | 132 (97.8) | 127 (94.8) | 132 (97.8) | 133 (100) |
| **Negative samples (n [%])^b^** | 3 (0.4) | 1 (1.4) | 0 (0) | 0 (0) | 0 (0) | 2 (1.5) | 0 (0) |
| **Contaminated samples (n [%])** | 14 (1.9) | 0 (0) | 3 (2.2) | 3 (2.2) | 7 (5.2) | 1 (0.7) | 0 (0) |
| **Median baseline (days)** | 4.08 | 3.83 | 4.63 | 4.02 | 4.00 | 4.17 | 4.08 |

^a^ Defined as a log10 CFU value below 1 ml^-1^.

^b^ Defined as a TTP longer than 42 days.

TTP: time-to-positivity; CFU: colony-forming unit.

## Table S2: Patient characteristics by rifampicin dose

| **Parameter** | | **Overall**  **(n = 83)** | **10 mg/kg**  **(n = 8)** | **20 mg/kg**  **(n = 15)** | **25 mg/kg**  **(n = 15)** | **30 mg/kg**  **(n = 15)** | **35 mg/kg**  **(n = 15)** | **40 mg/kg**  **(n = 15)** |
| --- | --- | --- | --- | --- | --- | --- | --- | --- |
| **Age, y** | | 31.0  (18.0-59.0) | 27.5  (19.0-49.0) | 27.0  (18.0-46.0) | 25.0  (19.0-46.0) | 40.0  (19.0-59.0) | 37.0  (21.0-59.0) | 34.0  (23.0-58.0) |
| **Weight, kg** | | 53.9  (40.2-84.2) | 56.85  (46.8-64.9) | 52.6  (41.8-62.7) | 52.8  (40.2-67.9) | 54  (45.7-84.2) | 57  (40.5-74) | 58.9  (46.7-64.8) |
| **BMI, kg/m^2^** | | 19.4  (14.7-30.9) | 20.5  (15.8-26.3) | 18.6  (16.8-26.2) | 19.3  (15.1-25.2) | 20.9  (16.4-30.9) | 19.4  (14.7-25.2) | 19.4  (17.2-24.9) |
| **Height, m** | | 1.66  (1.5-1.89) | 1.705  (1.53-1.77) | 1.63  (1.53-1.73) | 1.64  (1.6-1.82) | 1.65  (1.58-1.78) | 1.69  (1.5-1.83) | 1.68  (1.55-1.89) |
| **HIV infection** | | 3  (3.6) | 0  (0) | 0  (0) | 0  (0) | 2  (13.3) | 1  (6.7) | 0  (0) |
| **Race^a^** | | | | | | | | |
|  | **Colored** | 45  (54.2) | 5  (62.5) | 8  (53.3) | 11  (73.3) | 6  (40.0) | 10  (66.7) | 5  (33.3) |
|  | **Black** | 38  (45.8) | 3  (37.5) | 7  (46.7) | 4  (26.4) | 9  (60.0) | 5  (33.3) | 10  (66.7) |

Data are median values (ranges) or number (%) of patients per group.

HIV: human immunodeficiency virus.

^a^ “Black” refers to African natives, while “Colored” refers to a population group genetically descended from Southeast Asia.

# Supplementary Figures


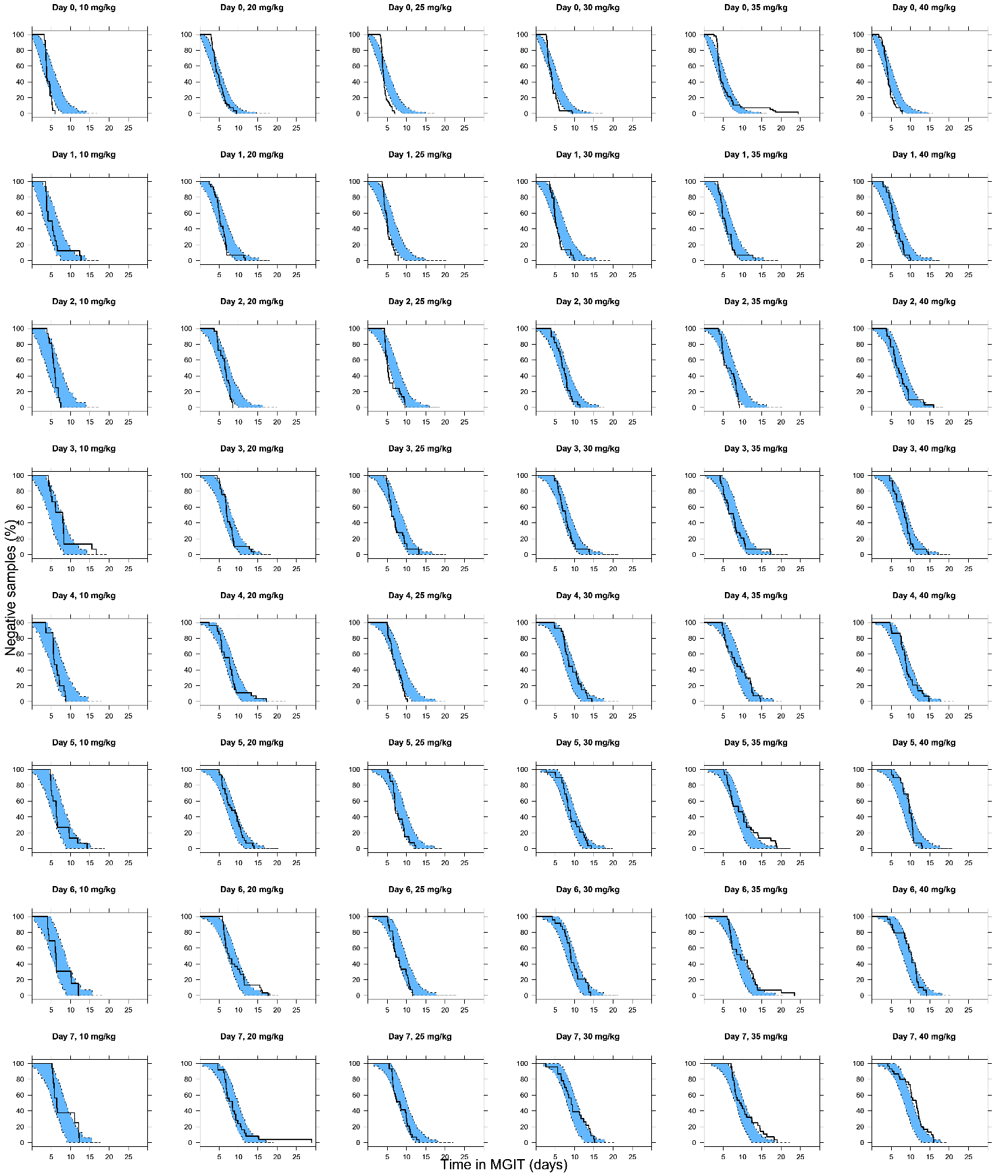


**Figure S1.** Visual predictive check of the final PK-CFU-TTP model describing time to produce a positive signal in the MGIT liquid culture, per day and rifampicin dose. Solid lines represent Kaplan-Meier (survival) curves based on the observed data. Shaded area outlines 95% prediction interval based on 1000 simulations using the model.
